# Strain‐based characterization of atrioventricular and left atrial remodelling in rat models of heart failure with preserved ejection fraction

**DOI:** 10.1113/EP093315

**Published:** 2026-02-17

**Authors:** Qingfeng Zhang, Wenhua Li, Hongmei Zhang, Yi Wang, Lixue Yin

**Affiliations:** ^1^ School of Medicine University of Electronic Science and Technology Chengdu China; ^2^ Ultrasound Medicine and Computational Cardiology Key Laboratory of Sichuan Province, Sichuan Provincial People's Hospital University of Electronic Science and Technology of China Chengdu China; ^3^ Department of Cardiovascular Ultrasound, Sichuan Provincial People's Hospital University of Electronic Science and Technology of China Chengdu China

**Keywords:** atrioventricular coupling, heart failure with preserved ejection fraction, hypertension, left atrium, speckle‐tracking echocardiography

## Abstract

Current experimental models of heart failure with preserved ejection fraction (HFpEF) lack standardized approaches for evaluating left atrial (LA) remodelling and atrioventricular (AV) coupling, leaving a critical gap in mechanistic understanding and phenotypic characterization. This study aimed to explore LA function and AV coupling status in a rat model of hypertension‐related HFpEF, thereby providing support for phenotype‐specific therapeutic strategies. We established two experimental rat models: A dual‐hit model (high‐fat diet combined with *N* ^ω^‐nitro‐l‐arginine methyl ester; HD + NAME) and a high‐salt‐sensitive model (Dahl salt‐sensitive; Dahl/SS). LA function and AV coupling were quantified using speckle‐tracking echocardiography (STE) with a modified LA imaging protocol and dedicated strain analysis software. Key parameters included phasic LA function, circumferential strain rates, LA stiffness index (LASI), and LA reservoir strain (LASr). Correlations with histopathological alterations were also examined. Both HFpEF groups exhibited significant left ventricular remodelling, diastolic dysfunction and reduced LASr compared with control (Control: 25.5 ± 3.4%, Dahl/SS: 17.8 ± 2.6%, and HD + NAME: 15.6 ± 2.9%; all *P* < 0.001 vs. Control). The HD + NAME model demonstrated higher LASI and *E*/early diastolic circumferential strain rate, indicating impaired AV coupling. Histological analysis revealed LA cardiomyocyte hypertrophy and interstitial fibrosis (fibrotic area: Control: 1.1 ± 0.5%; HD + NAME: 4.9 ± 1.7%, Dahl/SS: 6.7 ± 1.9%; all *P* < 0.001 vs. Control). Importantly, LASI correlated strongly with cardiomyocyte hypertrophy (*r* = 0.0.635) and fibrosis (*r* = 0.733; all *P* < 0.001). Standardized STE enables high‐resolution quantification of LA function and AV coupling in preclinical HFpEF models. Comparative evaluation of dual‐hit and salt‐sensitive hypertension models provides phenotypic stratification and yields novel mechanistic insights into atrioventricular decoupling in HFpEF.

## Abstract (highlights)

- **What is the central question of this study?**
  Can standardized methods be obtained for assessing left atrial (LA) remodelling and atrioventricular (AV) coupling in heart failure with preserved ejection fraction (HFpEF)?
- **What is the main finding and its importance?**
  With a rat‐specific speckle‐tracking echocardiography protocol optimized for LA imaging, LA stiffness index (LASI) and *E*/early diastolic circumferential strain rate (CSRe) were identified as robust biomarkers of AV uncoupling, establishing a new standard for evaluating LA function in preclinical HFpEF models. Combined use of LASI and CSRe within a unified diagnostic framework improved phenotypic discrimination between distinct HFpEF models and enhanced accuracy in monitoring cardiac functional changes.

## 1 INTRODUCTION

Heart failure with preserved ejection fraction (HFpEF) is a heterogeneous syndrome with complex pathophysiology, in which left atrial (LA) remodelling and impaired atrioventricular (AV) adaptation constitute pivotal features. However, LA dysfunction and AV uncoupling remain insufficiently studied, despite their potential role as key mechanisms driving HFpEF heterogeneity under haemodynamic stress. Although LA dysfunction is associated with adverse outcomes, clinical studies are constrained by limited tissue accessibility, absence of early markers, and uncontrolled phenotypic variability (Smiset et al., 2021; Yamamoto et al., 2014). Preclinical models are therefore essential to enable standardized assessment and to uncover early mechanistic pathways (Kagami et al., 2024; Valero‐Muñoz et al., 2017). Diastolic dysfunction in HFpEF imposes chronic mechanical stress on the LA, promoting progressive reservoir dysfunction, increased stiffness and elevated pulmonary venous pressures, which in turn contribute to impaired right ventricle–pulmonary artery coupling and clinical decompensation (Yuan et al., 2023; Kupczyńska et al., 2021). In recent years, the concept of atrial cardiomyopathy has gained attention, referring to structural, electrical and mechanical remodelling of the LA that may occur independently of, or in conjunction with, ventricular dysfunction (Li et al., 2022). Despite these insights, few studies have systematically combined pathophysiological evidence with advanced imaging modalities, such as speckle‐tracking echocardiography (STE). STE offers advantages over conventional volumetric indices, including higher sensitivity, better reproducibility and angle‐independence, to characterize atrial remodelling and AV uncoupling across distinct HFpEF phenotypes (Sinha et al., 2020; Leiner, 2022). Moreover, while clinical studies have documented LA–AV decoupling, its histological correlates and mechanistic heterogeneity remain poorly defined, largely due to limited access to myocardial tissue and early‐stage disease markers in human subjects.

To address this knowledge gap, we employed two well‐established hypertension‐related HFpEF rat models: (i) Dahl salt‐sensitive (Dahl/SS) rats, representing salt‐driven hypertension, and (ii) a dual‐hit model combining high‐fat diet with *N* ^ω^‐nitro‐l‐arginine methyl ester (HD + NAME), representing metabolic and endothelial stress (Barandiarán Aizpurua et al., 2018; Schiattarella et al., 2019; Withaar et al., 2021). These models capture distinct pathophysiological drivers of HFpEF, yet preclinical platforms that reliably recapitulate phenotype‐specific LA‐AV uncoupling remain scarce. Notably, few studies have directly compared the atrial functional and structural trajectories arising from divergent hypertensive stressors, limiting mechanistic understanding of LA dysfunction subtypes. We hypothesized that different underlying aetiologies of HFpEF would result in LA functional impairment and pathological remodelling, and that these differences could be non‐invasively quantified using an optimized LA‐STE protocol. In this study, we integrated an optimized LA‐targeted STE protocol with histopathological assessment and exercise capacity testing to delineate functional heterogeneity across HFpEF phenotypes. We further sought to validate imaging biomarkers such as the LA stiffness index (LASI) and diastolic circumferential strain rate (CSRe), and to elucidate their relationships with atrial pathological remodelling. The overarching objective was to identify non‐invasive biomarkers for phenotypic stratification, thereby enhancing the clinical relevance of our findings.

## 2 METHODS

### 2.1 Ethical approval

All experimental procedures were approved by the Institutional Ethics Committee (Approval Number: Research 2023−341) and conducted in accordance with the ARRIVE guidelines and the Guide for the Care and Use of Laboratory Animals. The research compiled with *Experimental Physiology*’s policies regarding animal experimentation.

### 2.2 Animal models

A total of 48 rats were included in this study, comprising 32 healthy male and female Sprague–Dawley rats (6–8 weeks old, 300–400 g) and 16 salt‐sensitive Dahl/SS rats (300–400 g), obtained from the Experimental Animal Centre of Sichuan Provincial People's Hospital. Rats were housed under a 12 h light/dark cycle in a specific pathogen‐free environment with ad libitum access to standard laboratory chow and water. Following a 1‐week acclimatization period, animals were randomized into three groups: Control, HD + NAME and Dahl/SS, with equal numbers of males and females (*n* = 8 per sex per group). HFpEF was induced in the Dahl/SS group by feeding rats an 8% NaCl diet for 10 weeks. In the HD + NAME group, Sprague–Dawley rats were fed a 60% high‐fat diet (D12492, Research Diets, Inc, USA) combined with drinking water containing N^ω^‐nitro‐l‐arginine methyl ester (l‐NAME, 0.5 g/L) for the same duration. Control animals received a standard diet and free access to water. Systolic blood pressure (SBP) was measured weekly using a non‐invasive tail‐cuff system (BP100A, Taimeng Software, China), with the sensor positioned 2 cm from the tail base. Venous blood samples were collected and centrifuged at 1000 × *g* for 10 min to separate the serum. The resulting serum was aliquoted and stored at −80°C until analysis. N‐terminal pro‐B‐type natriuretic peptide (NT‐proBNP) levels were measured using a commercially available enzyme‐linked immunosorbent assay (ELISA) kit according to the manufacturer's instructions (Elabscience Biotechnology Co, China). At the end of the study, body weight, tibial length and heart weight were recorded, and the heart weight‐to‐tibial length (HW/TL) ratio was calculated as an index of cardiac hypertrophy.

### 2.3 Echocardiography

Transthoracic echocardiography was performed using a high‐resolution imaging system (Vivid E95, GE Healthcare, Horten, Norway) equipped with a 12S high‐frequency convex array transducer (8–14 MHz). Rats were positioned in the supine position on a temperature‐controlled platform following chest depilation. Anaesthesia was induced with inhaled 2–3% isoflurane in oxygen, and heart rate was maintained between 300 and 350 beats/min to ensure haemodynamic stability. Standard two‐dimensional and Doppler images were acquired from parasternal long‐axis, parasternal short‐axis (at the papillary muscle level) and apical four‐chamber views. A modified apical view was obtained by tilting the probe slightly leftward and upward from the conventional LV outflow tract orientation to optimize LA visualization. Images were acquired at ≥173 frames per second, capturing at least six consecutive cardiac cycles for offline speckle‐tracking analysis. Mitral inflow velocities (*E* and *A* waves) were measured using pulsed‐wave Doppler in the apical four‐chamber view. Tissue Doppler imaging at the septal annulus was used to measure early diastolic mitral annular velocity (*e*′). *E*/*A* and *E*/*e*′ ratios were calculated as indices of LV diastolic function (Huc et al., 2018; Litwin et al., 1995). Left ventricular ejection fraction (LVEF) was calculated from long‐axis views using the biplane method of disks. Relative wall thickness (RWT) and left ventricular mass (LVMASS) were automatically derived from M‐mode and two‐dimensional measurements in accordance with established echocardiographic guidelines.

### 2.4 Optimization of LA Imaging

**FIGURE 1:**
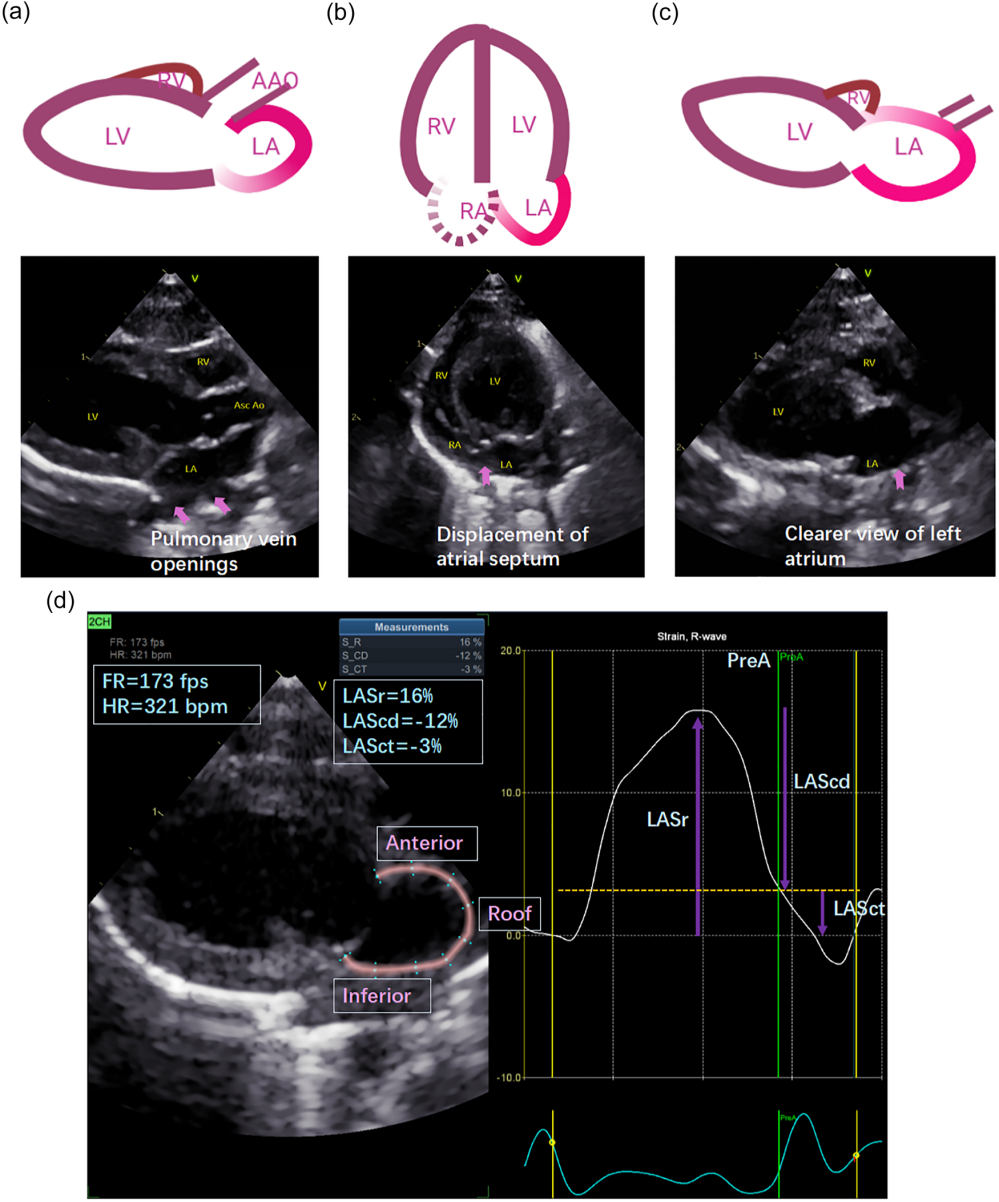
(a–c) Different views of the left atrium. (a) left ventricular long‐axis view, displaying an irregularly shaped with visible pulmonary vein openings. (b) Four‐chamber view, where LA visualization is obscured due to significant displacement of the atrial septum. (c) Modified LA view, achieved by slightly shifting the probe to the left, providing improved imaging clarity. (d) Example of three phases of LA strain analysis in HFpEF rat. The software automatically identifies the pre‐atrial contraction phase. The analysis is performed using dedicated LA strain analysis software, providing enhanced accuracy and reliability compared to conventional strain techniques. FR = 173 fps. AAo, ascending aorta; FR, frame rate; HFpEF, heart failure with preserved ejection fraction; HR, heart rate; LA, left atrial; LV, left ventricular; RA, right atrial; RV, right ventricular.

In clinical practice, LA volume and strain are typically assessed using apical four‐chamber and two‐chamber views. However, replicating these imaging planes in rodents is technically challenging due to anatomical variability and operator‐dependent inconsistency. To address these limitations, we optimized the LA imaging strategy by using a modified parasternal long‐axis view combined with a smaller ultrasound transducer, higher frame rates and improved probe manoeuvrability. This approach provided superior LA visualization by minimizing interference from the atrial septum and pulmonary veins, thereby facilitating more accurate and reproducible functional assessments (Hanif et al., 2017; Shen et al., 2022). Traditional LA imaging in rodents is often confounded by artifacts from pulmonary venous inflow and atrial septal motion, which can compromise strain analysis (Figure 1a–c). Representative LA strain maps and corresponding strain curves are shown in Figure 1d.

### 2.5 LA phasic strain and AV function analysis

In the modified apical imaging view, LA strain was quantified using the functional imaging technique, with manual contour corrections applied as necessary. The strain curve was derived by tracking the endocardial border from the anterior mitral annulus along the lateral wall and roof to the inferior mitral annulus. Efforts were made to minimize interference from thin atrial walls, pulmonary vein insertions, and the LA appendage. Image quality and frame rate optimization were prioritized, as accurate LA analysis requires high temporal resolution. Previous studies have recommended ≥40 frames per second (fps) (Lindner, 2023). In the present study, a frame rate of 173 fps was consistently achieved, allowing precise quantification of LA strain and improving the reliability of HFpEF model evaluation. LA strain was analysed with the R–R interval as temporal reference, with end‐diastole set as the zero baseline. Conduit and contraction strains were expressed as negative values. LASI was calculated as the ratio of *E*/*e*′ to LA reservoir strain (*E*/*e*′/LASr). Circumferential strain rates (CSR) were assessed at three phases: systolic (CSRs), early diastolic (CSRe) and isovolumetric relaxation (CSRivr) circumferential strain rate. The AV compliance index (*E*/CSRe) was also calculated for group comparisons. All analyses were independently performed by two experienced echocardiographers who were blinded to group allocation, ensuring objectivity and reproducibility.

### 2.6 Exercise endurance testing

Exercise endurance was assessed using a rodent‐specific treadmill system (SA101B, SansBio, Jiangsu SansBio Biotechnology Co, China). Rats underwent a standardized acclimation protocol on the two consecutive days preceding the formal exercise test. The treadmill was set to a walking speed of 5 m/min for 10 min and then progressively increased up to 10 m/min, for a total exercise duration of 20 min of exercise with 10 grade. Rats were fasted for 4–6 h before treadmill running to minimize variability related to recent feeding. The protocol began at a speed of 5 m/min for 5 min, followed by 10 m/min at a 15° incline for 10 min, 15 m/min for 20 min, and finally 20 m/min until exhaustion. Exhaustion was defined as (i) inability to maintain treadmill pace after >20 electrical stimuli, or (ii) immobility under continuous shock for >5 s. The gradual increase in intensity ensures a measurable submaximal phase while ultimately driving the animal to volitional exhaustion, providing an integrated assessment of cardiopulmonary and musculoskeletal function (Pet et al., 2016; Salzano et al., 2021). Total running distance and exercise duration were recorded as indices of endurance.

### 2.7 Histopathological evaluation

Rats were humanely euthanized under deep anaesthesia by exsanguination via cardiac puncture. Following median sternotomy, hearts were excised and the left atrium was carefully dissected and processed for histological analysis. LA tissue was fixed, paraffin‐embedded, and sectioned for staining. Interstitial fibrosis was quantified using Masson's trichrome staining, while cardiomyocyte hypertrophy was evaluated with wheat germ agglutinin (WGA) staining. For each group, at least 10 randomly selected fields at ×400 magnification were captured from each section, ensuring that the tissue filled the entire field and that background illumination remained consistent across images. Images were analysed using Image‐Pro Plus 6.0, and the mean cell area per section was calculated. Quantitative histological assessments were performed to correlate structural remodelling with functional parameters.

### 2.8 Statistical analysis

All statistical analyses were performed using SPSS 26.0 (IBM Corp., Armonk, NY, USA) and GraphPad Prism 10.0 (GraphPad Software, Boston, MA, USA). Data are presented as means ± standard deviation (SD). Normality was assessed using the Shapiro–Wilk test. Comparisons among the three groups (Control, Dahl/SS, and HD + NAME) were performed using one‐way analysis of variance (ANOVA) followed by Bonferroni *post hoc* correction for multiple comparisons. For comparisons between two groups (for correlation or subgroup analyses), Student's unpaired *t*‐test was applied. Correlations between continuous variables were evaluated using Pearson's method. To assess potential sex‐related effects, two‐way ANOVA was conducted with ‘group’ and ‘sex’ as fixed factors. No significant main effect of sex or group × sex interaction was observed (*P* > 0.1 for any of the key parameters); therefore, male and female data are presented separately for descriptive purposes but were analysed independently to minimize confounding. Statistical significance was defined as *P *< 0.05.

## 3 RESULTS

### 3.1 Conventional echocardiographic parameters

**FIGURE 2:**
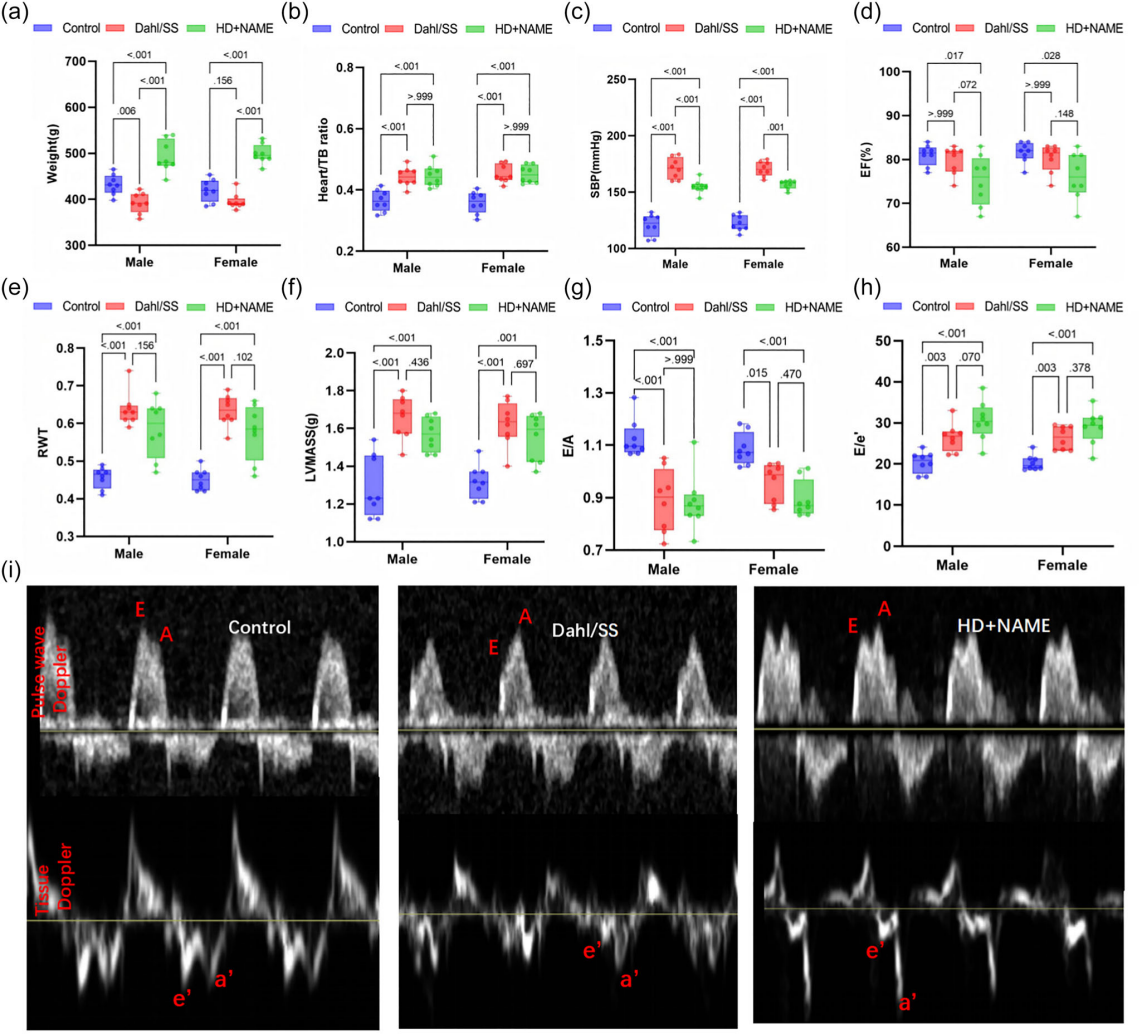
(a–h) Basic characteristics and conventional echocardiographic parameters in a comparison of Control, Dahl/SS and HD + NAME groups. (a) Body weight; (b) heart weight normalized to tibia length; (c) SBP; (d) EF; (e) RWT; (f) LVMASS; (g) the ratio of early and late mitral flow velocity (*E*/*A*); (h) the ratio of early mitral flow velocity and diastolic septal mitral annulus velocity (*E*/*e*′). (i) Mitral inflow spectrum and tissue Doppler in assessing diastolic function in the three groups. EF, ejection fraction; LVMASS, left ventricular mass; RWT, relative wall thickness; SBP, systolic blood pressure; TB, tibia.

Body weight was significantly higher in the HD + NAME group compared with control (471 ± 32 g vs. 422 ± 39 g, *P* < 0.001, Figure 2a), while the Dahl/SS group showed lower weight (394 ± 29 g, P = 0.006). Despite these systemic differences, both HFpEF models exhibited pronounced cardiac hypertrophy, as reflected by increased heart weight‐to‐tibia length ratios (Dahl/SS: 0.42 ± 0.06; HD + NAME: 0.44 ± 0.05; all *P* < 0.001 vs. control: 0.36 ± 0.04, Figure 2b). SBP was markedly elevated in both HFpEF groups (Dahl/SS: 168 ± 11 mmHg; HD + NAME: 155 ± 10 mmHg) compared with control (122 ± 12 mmHg, all *P* < 0.001 vs. control), confirming hypertension‐induced cardiac stress (Figure 2c). As expected, all HFpEF model groups exhibited preserved LVEF compared with control (Figure 2d). Both Dahl/SS and HD + NAME rats exhibited significantly increased RWT and LVMASS compared to control (all *P* < 0.001 vs. control, Figure 2e, f). Diastolic dysfunction was supported by elevated *E*/*e*′ ratios (P < 0.001) and reduced *E*/*A* ratios (Control: 1.12 ± 0.07; Dahl/SS: 0.86 ± 0.05; HD + NAME: 0.89 ± 0.04; P < 0.001) (Figure 2g, h). Heart rate was continuously monitored during echocardiographic image acquisition, and no significant intergroup differences were observed (*P* = 0.470).

### 3.2 Assessment of LA strain and AV function

**FIGURE 3:**
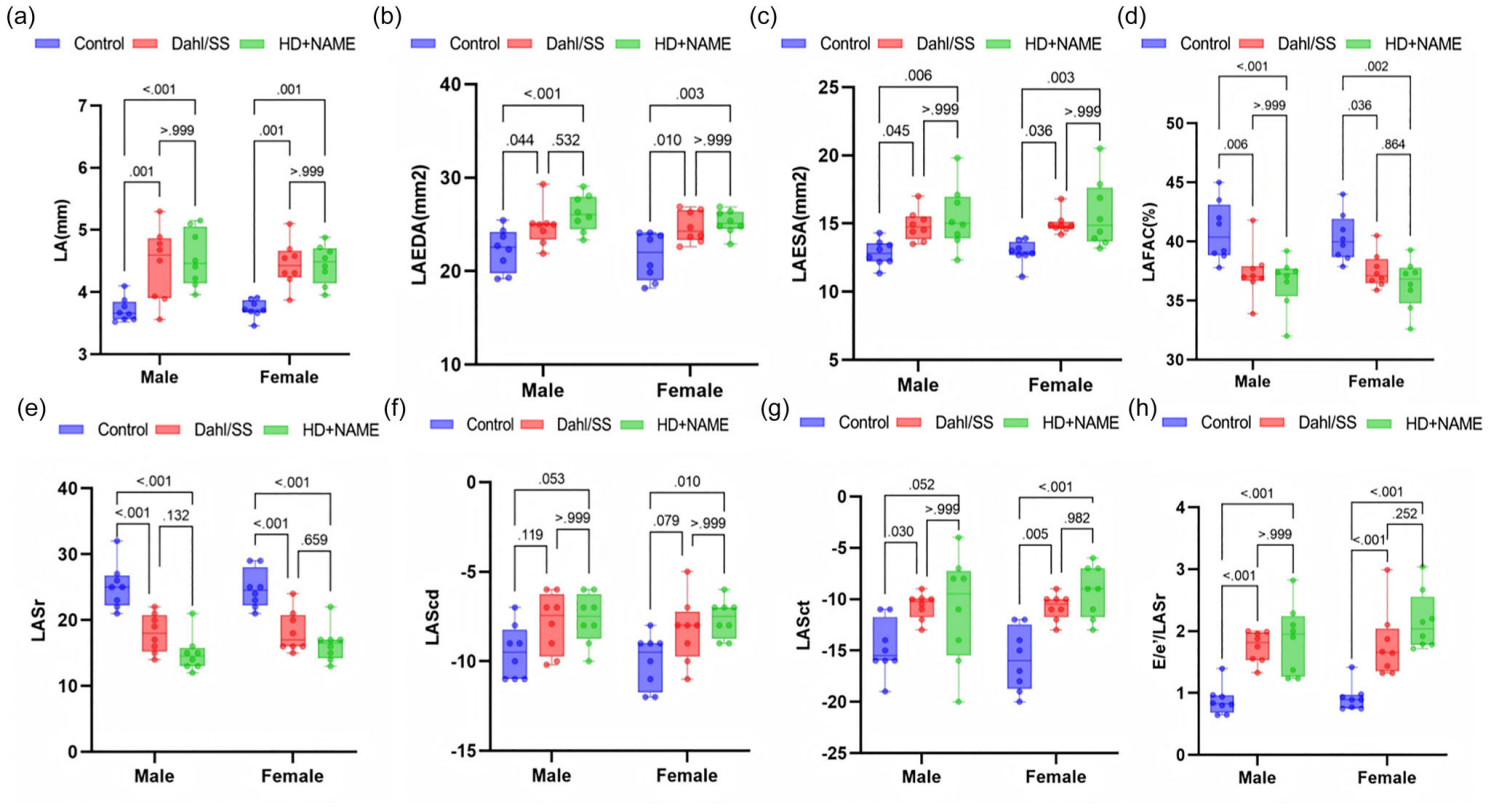
Echocardiographic assessment of LA function across different phases showing parameters (a) LA diameter; (b) LAEDA; (c) LAESA; (d) LAFAC; (e) LASr during systole; (f) LAScd during early diastole; (g) LASct during late diastole; (h) LASI (*E*/*e*′/LASr). LA, left atrial; LAEDA, LA end‐diastolic area; LAESA, LA end‐systolic area; LAFAC, LA fractional area change; LAScd, LA conduit strain; LASct, LA contraction strain; LASr, LA reservoir strain; LASI, LA stiffness index.

Structural and functional analyses revealed distinct patterns of atrial remodelling across the two HFpEF models (*n* = 24). Both Dahl/SS and HD + NAME male rats exhibited mild LA dilation compared to control (anteroposterior diameter: Control, 3.7 ± 0.6 mm; Dahl/SS, 4.4 ± 0.5 mm; HD + NAME, 4.4 ± 0.7 mm; with no difference between the two model groups, *P = *0.07, Figure 3a). Model‐dependent alterations in LA phasic function were observed. Notably, both HFpEF groups demonstrated enlarged LA end‐systolic area (LAESA) (Figure 3c) and reduced LA fractional area change (LAFAC) (Figure 3d), indicating impaired LA contractile performance. STE revealed preserved conduit function with no statistical significance (LAScd: Control, −9.8 ± 2.1%; vs. Dahl/SS, −8.0 ± 1.8%, *P *= 0.119; vs. HD + NAME, −7.6 ± 2.3%, *P = *0.053, Figure 3f), but significant impairments in reservoir functions. Compared with control, LASr was markedly reduced (Control, 25.5 ± 3.4%; Dahl/SS, 17.8 ± 2.6%, HD + NAME, 15.6 ± 2.9%; all *P* < 0.001 vs. control, Figure 3e). Importantly, LASI emerged as a sensitive marker of AV dysfunction, demonstrating significant increases in both HFpEF models (Control, 0.92 ± 0.25; Dahl/SS 1.7 ± 0.29; HD + NAME, 1.9 ± 0.34, all *P *< 0.001 vs. control; Figure 3h).

**FIGURE 4:**
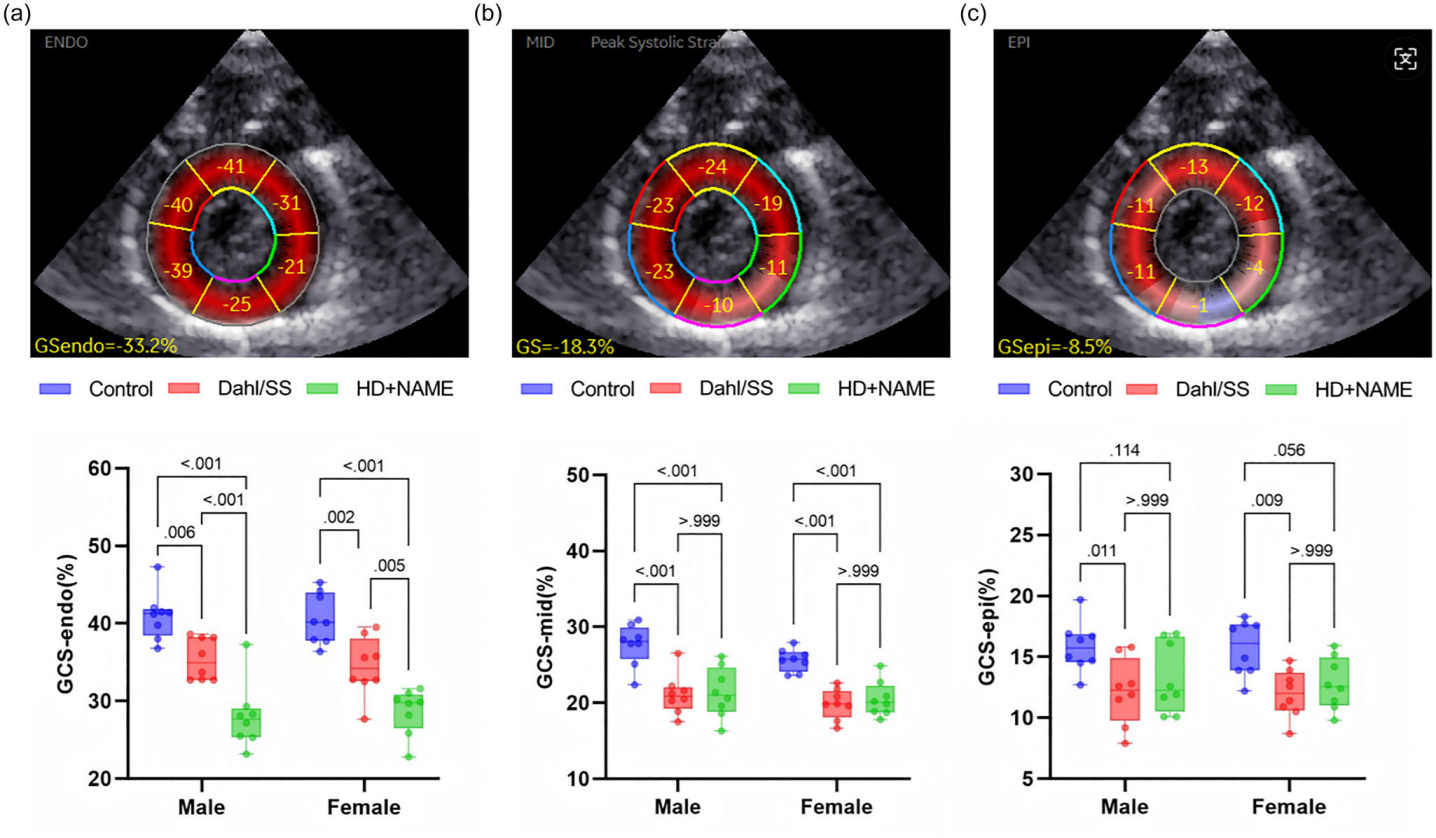
Representative images and comparison of circumferential layer‐specific strain. (a) GCS‐endocardial strain, GCS‐endo; (b) GCS‐mid‐myocardial strain, GCS‐mid; (c) GCS‐epicardial strain, GCS‐epi. GCS, global circumferential strain.

**FIGURE 5:**
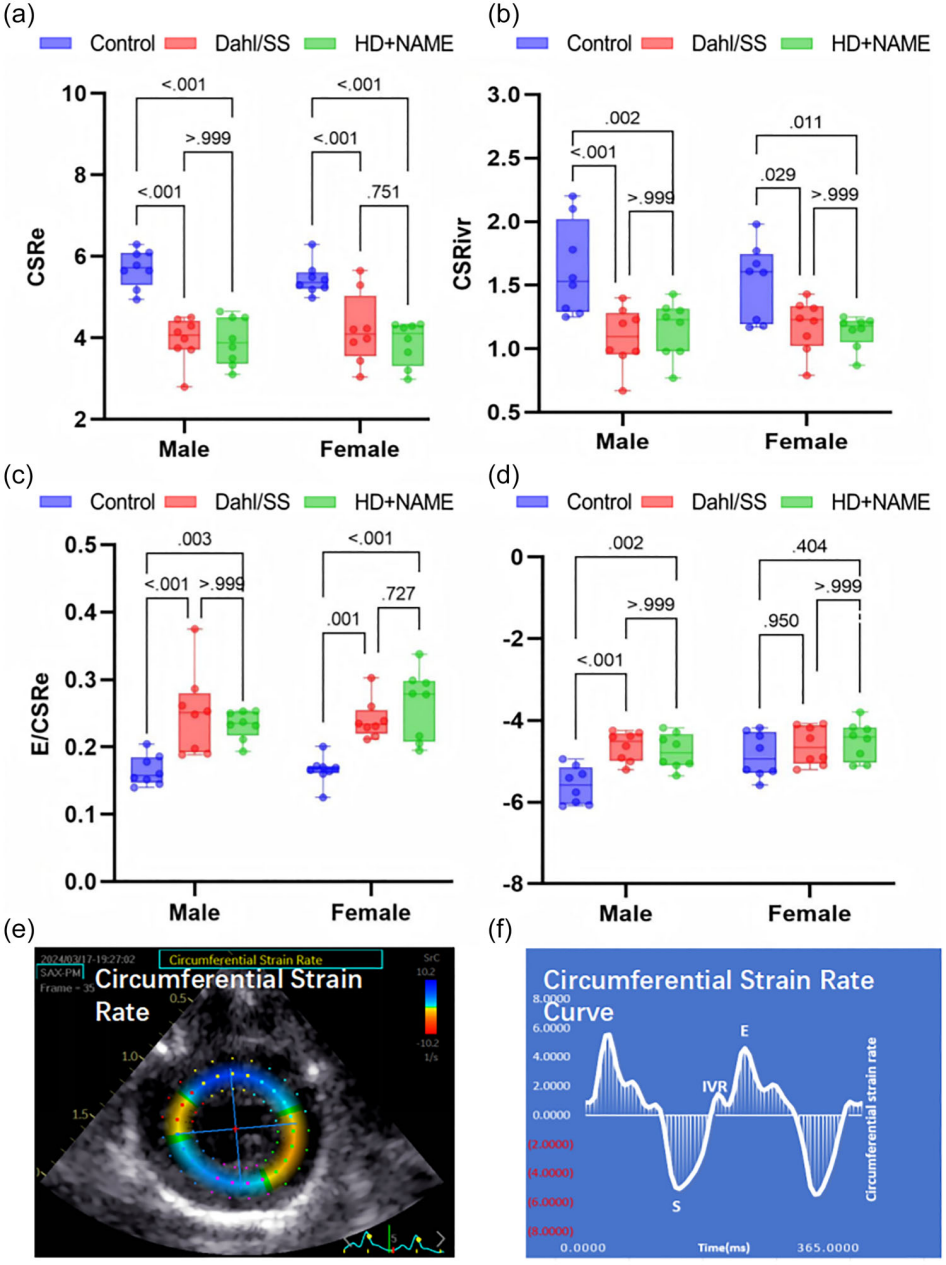
Comparison of LV CSR. (a–d) Graphs showing parameters: (a) CSRe; (b) CSRivr; (c) atrioventricular compliance index (*E*/CSRe) as the ratio of *E* to CSRe; (d) CSRs. (e) Representative image in HFpEF. (f) Typical curve of CSR in HFpEF. CSR, circumferential strain rate; CSRe, early diastolic circumferential strain rate; CSRivr, isovolumetric relaxation circumferential strain rate; CSRs, systolic circumferential strain rate; *E*, early diastolic; HFpEF, heart failure with preserved ejection fraction; IVR, isovolumic relaxation; LV, left ventricular; S, systolic.

Circumferential layer‐specific strain (CS), a novel parameter validated in preclinical models for assessing transmural myocardial mechanics, was significantly reduced in both HFpEF groups compared with control (*P* < 0.001, Figure 4a–c). A characteristic gradient of strain impairment was observed, with endocardial CS showing the most pronounced reduction (Control, −41.6 ± 4.3%; vs. Dahl/SS, −34.2 ± 3.1%, *P *= 0.006; vs. HD + NAME, −29.5 ± 3.8%, *P *< 0.001; Figure 4a), closely paralleling the endocardial‐to‐epicardial strain attenuation reported in human HFpEF cohorts (Frimodt‐Møller et al., 2024). Regional segmentation of each myocardial layer into six sectors (18 total) further revealed the HFpEF‐related remodelling. To complement strain analysis, CSR parameters were evaluated across systolic (CSRs), isovolumic relaxation (CSRivr) and early diastolic (CSRe) phases (Murai et al., 2017). CSRs remained preserved across groups (*P *= 0.135, Figure 5a); however, both CSRivr and CSRe were significantly reduced in HFpEF rats (Figure 5b, d). To further characterize AV coupling, we calculated the *E*/CSRe ratio, a load‐sensitive index of compliance (Wang et al., 2007). This ratio was significantly elevated in both HFpEF groups (Control group, 0.16 ± 0.06 vs. Dahl/SS, 0.25 ± 0.06, *P *< 0.001; vs. HD + NAME, 0.23 ± 0.05, *P *= 0.003) compared to control (*P* < 0.001, Figure 5c).

### 3.3 Maximal exercise endurance testing and correlation analysis

**FIGURE 6:**
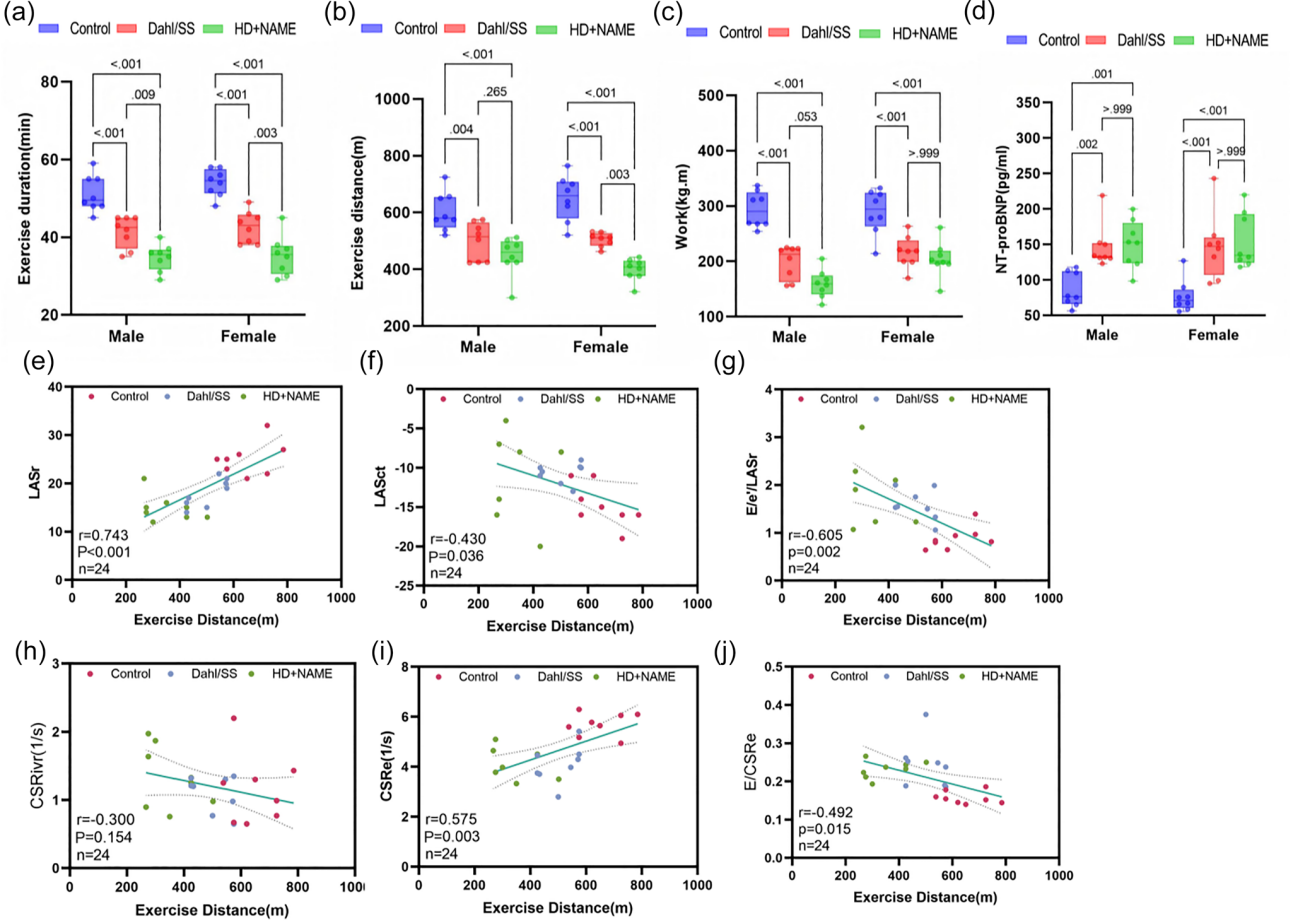
(a–c) Exercise capacity and its correlations with echocardiographic parameters. Comparisons of exercise performance among groups, including (a) exercise duration, (b) distance, and (c) body weight–normalized work. (d) Comparison of NT‐proBNP levels between the two groups of model animals. (e–j) Correlation analyses of exercise distance with (e) LASr, (f) LASct, (g) *E*/*e*′/LASr, (h) CSRivr, (i) CSRe, (j) *E*/CSR. CSR, circumferential strain rate; CSRe, early diastolic circumferential strain rate; CSRivr, isovolumetric relaxation circumferential strain rate; LASct, left atrial contraction strain; LASr, left atrial reservoir strain.

Exercise endurance was assessed using a standardized treadmill protocol, with performance quantified by running distance, exercise duration, and total work (kg m) normalized to body weight (Pluteanu et al., 2018; Reil et al., 2016). Both HFpEF models demonstrated significantly impaired exercise endurance compared with control, with the HD + NAME group exhibiting the most severe limitation (Figure 6a, b). After adjustment for body weight, however, exercise work did not differ significantly between the Dahl/SS and HD + NAME groups (Figure 6c). Moreover, NT‐proBNP was significantly elevated in both model groups (Figure 6d). Importantly, the pattern of reduced exercise tolerance aligned with echocardiographic evidence of left atrial–ventricular uncoupling and diastolic dysfunction (Figure 6e–j).

### 3.4 Correlation of LA strain and AV compliance with cardiomyocyte hypertrophy and fibrosis

**FIGURE 7:**
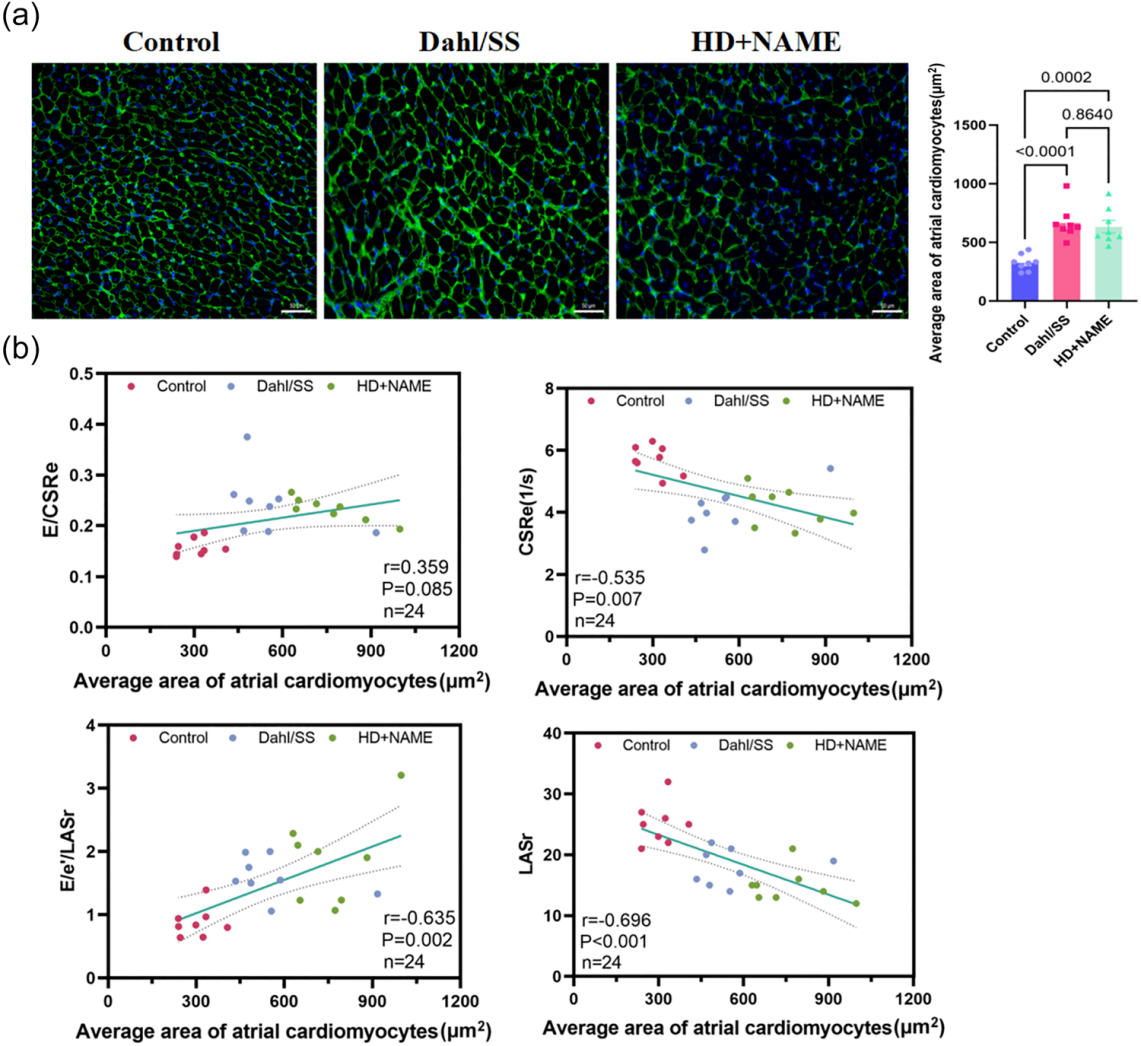
(a) Correlation analysis of LA strain and atrioventricular compliance with LA cardiomyocyte hypertrophy WGA staining; scale bar: 50 µm. (b) Correlation analyses of LASr, LASI, CSRe and *E*/CSRe with LA cardiomyocyte hypertrophy. CSRe, early diastolic circumferential strain rate; LA, left atrial; LASI, LA stiffness index; LASr, LA reservoir strain; WGA, wheat germ agglutinin.

**FIGURE 8:**
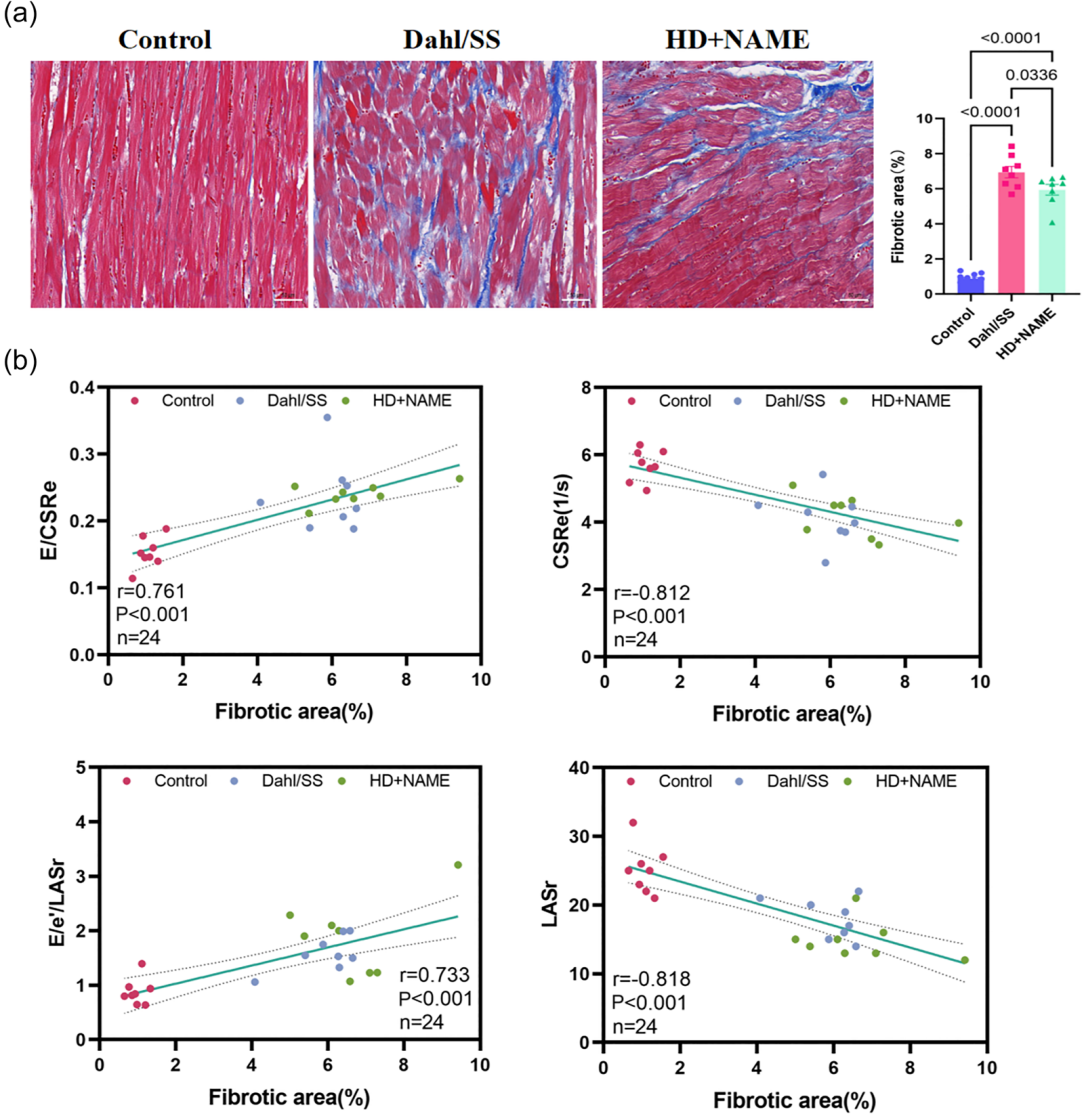
(a) Correlation analysis of LA strain and atrioventricular compliance with LA tissue fibrosis Masson's trichrome staining; scale bar: 50 µm. (b) Correlation analyses of LASr, LASI, CSRe and *E*/CSRe with LA fibrosis. CSRe, early diastolic circumferential strain rate; LA, left atrial; LASI, LA stiffness index; LASr, LA reservoir strain.

To explore the structural basis of atrial dysfunction in HFpEF, high‐resolution fluorescence imaging (Leica AF 6000) was performed using dual‐staining protocols: WGA to delineate cardiomyocyte membranes and Masson's trichrome to visualize interstitial collagen deposition. Quantitative morphometric analysis revealed significant cardiomyocyte hypertrophy, with cross‐sectional area nearly doubled in both HFpEF models (Dahl/SS: 687  ± 133 µm^2^; HD + NAME: 573 ± 156 µm^2^) compared with control (345 ± 101 µm^2^; all *P *< 0.001) (Figure 7a). Fibrotic burden was also significantly increased (Control: 1.1 ± 0.5%; Dahl/SS: 6.7 ± 1.9%; HD + NAME: 4.9 ± 1.7%; all *P* < 0.001 vs. control) (Figure 8a). Importantly, echocardiographic indices of LA and AV function, including LASr, LASI, CSRe and E/CSRe, correlated strongly with histopathological remodelling (Figures 7, 8). Among these, LASI exhibited the strongest associations, correlating positively with cardiomyocyte hypertrophy (*r* = 0.635, *P* = 0.002) and fibrosis burden (*r* = 0.733, *P* < 0.001), while LASr showed inverse correlations (hypertrophy: *r* = −0.696, *P* < 0.001; fibrosis: *r* = −0.818, *P* < 0.001).

## 4 DISCUSSION

Our study provides several key insights. First, we established a rat‐specific STE protocol optimized for LA imaging, enabling precise quantification of atrial mechanics. Using this approach, we identified LASI and *E*/CSRe as robust biomarkers of AV uncoupling, both strongly correlated with histopathological remodelling. These parameters represent reliable, non‐invasive markers of atrial dysfunction and establish a new standard for evaluating LA function in preclinical HFpEF models. Second, the combined use of LASI and CSRe within a unified diagnostic framework improved phenotypic discrimination between distinct HFpEF models and enhanced accuracy in monitoring cardiac functional changes.

The evaluation of LA structure and function has gained increasing prominence in HF management due to its strong association with disease progression and clinical symptom burden (Shuai et al., 2023). Nevertheless, standardized methodologies and consensus guidelines for assessing LA function remain lacking, particularly in experimental HFpEF models. Most prior studies have relied on volumetric or diameter‐based indices (Vianna‐Pinton et al., 2009), whereas STE has emerged as a powerful imaging modality for LA strain assessment owing to its high reproducibility and angle independence, as demonstrated by Sirbu et al. (2006) and Bode et al. (2021). By enabling detailed quantification of atrial mechanics, STE provides superior spatial resolution and more refined functional assessment than conventional echocardiographic techniques (Qiu et al., 2021; Tsang et al., 2006). However, methodological challenges remain. Existing approaches to rat LA strain analysis frequently rely on LV‐oriented software, which introduces technical limitations and potential inaccuracies (Zhang et al., 2023; Cameli et al., 2016; Vieira et al., 2014). To address this, we adapted functional imaging specifically for LA strain assessment, optimizing contouring for the thin atrial septum while minimizing artifacts from pulmonary veins and the LA appendage. This refinement not only ensures the robustness of our findings but also improves the precision of LA function evaluation in small‐animal HFpEF models.

In the HFpEF rat model, we observed significant reductions in LA strain during both the reservoir and active contraction phases, accompanied by markedly elevated LASI and without discernible sex differences. The association between LA dysfunction and HFpEF severity is well established, with growing recognition of AV compliance as a pivotal determinant of disease progression (Zakeri et al., 2016; Zhang et al., 2024). Recent clinical studies (Backhaus et al., 2023; Fortuni et al., 2024) have demonstrated that STE‐derived LA strain parameters reliably distinguish invasively confirmed HFpEF from non‐cardiogenic dyspnoea. Reddy et al. (2020) further reported substantial impairments in reservoir and conduit strains, which were strongly predictive of abnormal haemodynamics, reduced exercise capacity and adverse outcomes in HFpEF patients. In addition, Nagueh et al. (2016) identified an LA contraction strain threshold of <33% as a highly sensitive (88%) and specific (77%) diagnostic marker for HFpEF, outperforming the 2016 European Society of Cardiology (ESC) criteria by 12%. Beyond diagnosis, STE has also emerged as a sensitive tool for detecting myocardial fibrosis, effectively differentiating hypertrophic cardiomyopathy from Fabry disease (Hussain et al., 2024; Monte et al., 2023; Krämer et al., 2013), with strain parameters demonstrating strong correlations with fibrosis burden. Mátyás et al. (2018) further reported that CSRe is closely associated with LV myocardial fibrosis in diabetic patients. To our knowledge, this is the first study to integrate LA strain indices within AV compliance into a hypertension‐related HFpEF diagnostic framework. This approach enhances phenotypic classification and aligns with emerging HFpEF subtypes dominated by LA myopathy, which are increasingly recognized in human cohorts, particularly in older individuals, women and patients with elevated atrial stiffness (Rogala et al., 2023). HFpEF is increasingly recognized as a heterogeneous clinical syndrome encompassing multiple pathophysiological phenotypes (Zeng & Chen, 2019). Importantly, the Dahl/SS and HD + NAME models recapitulate this phenotype through distinct hypertensive and metabolic stress pathways, highlighting the necessity of phenotype‐specific diagnostic and therapeutic strategies. The two HFpEF models were selected to represent distinct aetiologies reported in the literature: the Dahl/SS model is characterized by salt‐sensitive hypertension with associated renal and inflammatory burden (Beyer et al., 2014), whereas the HD + NAME model recapitulates features of nitric oxide deficiency, vascular stiffness and microvascular dysfunction (Sanhueza‐Olivares et al., 2026). Histopathological assessment (Figures 7, 8) revealed that both models exhibited increased cardiomyocyte cross‐sectional area and interstitial collagen deposition, indicative of myocardial hypertrophy and fibrosis, and with more pronounced fibrotic remodelling in the left atrium of Dahl/SS rats. Accordingly, the analysis aimed to determine whether these divergent remodelling patterns would translate into distinct LA functional phenotypes detectable using the STE protocol.

Beyond haemodynamic derangements, LA remodelling in HFpEF is shaped by myocardial hypertrophy, abnormal titin phosphorylation, and microvascular rarefaction, even in the absence of chronic atrial hypertension or overt LA fibrosis (Rosas et al., 2024). Defining LA function in preclinical models is therefore essential for early HFpEF detection and the development of targeted interventions. In the early stages of hypertension‐induced HFpEF, compensatory LA mechanisms help preserve LV filling during the reservoir and contraction phases, thereby buffering the pulmonary circulation (Rossi et al., 2018). However, as the disease progresses, increased LV end‐systolic stiffness and elevated diastolic pressures surpass the compensatory capacity of the left atrium, resulting in impaired contractility, worsening pulmonary hypertension, and the development of maladaptive remodelling, often referred to as LA myopathy (Seferović et al., 2019). This maladaptive state promotes right ventricular dysfunction, functional mitral regurgitation due to annular dilation, and ultimately poor clinical outcomes in HFpEF (Wang et al., 2024; Rosenkranz et al., 2016).

Our study emphasizes the role of LA remodelling and AV decoupling in the evaluation of hypertension‐related HFpEF models, while also addressing potential sex‐specific differences. Although prior clinical studies have demonstrated that elderly female patients often exhibit disproportionately larger LA volumes relative to LV volumes, our findings revealed no significant sex differences or interaction effects, reinforcing age as a dominant determinant of LA dysfunction in HFpEF (Hoshida et al., 2018). Consistent with previous reports, our data support LASI as a superior prognostic marker compared with isolated LV filling pressure indices for predicting adverse outcomes in HFpEF patients (Kim et al., 2023; Dang et al., 2024). Impairments in LA reservoir and contractile function, reflected by elevated LASI and *E*/CSRe, highlight the mechanistic importance of AV uncoupling. Shortened isovolumic relaxation time (IVRT) and increased *E*/*A* ratio further emphasize the necessity of multiparametric evaluation for accurate disease characterization. Together, these findings demonstrate that combined assessment of LA strain and CSRe within a unified diagnostic framework enhances HFpEF phenotyping and risk stratification. Notably, both LA and LV functional impairments were already evident at 10 weeks in our HFpEF models. Mechanistically, salt‐sensitive HFpEF is primarily driven by hypertension, with SBP in Dahl/SS rats exceeding that of HD + NAME rats by ∼20 mmHg, thereby promoting atrial remodelling. By contrast, the HD + NAME model disrupts nitric oxide‐dependent vascular relaxation, with the progression of atrial myopathy driven by mitochondrial dysfunction and oxidative stress, ultimately contributing to diastolic impairment and atrial remodelling (Kuczeriszka & Wąsowicz, 2022; Wang et al., 2022). Although the observed linear relationships were statistically significant, the relatively low coefficients of determination (*r* ^2^) indicate that only a modest proportion of the variance in LA strain parameters can be explained by the histopathological changes. Comparative studies in other fibrosis‐driven models, such as aortic constriction or angiotensin‐II infusion, indicate that atrial fibrillation (AF) occurs only in cases of extensive fibrosis (approximately four‐fold increase vs. control) (Schnelle et al., 2018). Given that atrial fibrosis and dysfunction often precede AF onset, our findings suggest that LASr and AV compliance may serve as valuable experimental endpoints in preclinical therapeutic studies.

### 4.1 Limitations

This study did not include invasive LV pressure–volume measurements or an in‐depth analysis of underlying molecular mechanisms. The preclinical model used young adult rats (approximately 20 weeks old), and the aetiology of HFpEF in these animals differs substantially from that observed in humans. Nonetheless, the HFpEF models exhibited characteristic echocardiographic and biochemical features, including elevated *E*/*e*′ ratios and increased NT‐proBNP levels, all corroborated by histopathological validation. The thin‐walled structure of the left atrium introduces potential variability in strain tracking; however, the modified LA imaging protocol used in this study prioritizes area‐based evaluations rather than geometry‐dependent volume calculations, thereby improving measurement reliability. Although anaesthesia can affect heart rate, standardized protocols were applied to maintain heart rates within the 300–350 bpm range, minimizing bias in R–R interval‐dependent strain analyses. Future investigations should incorporate AF models to better characterize atrial myopathy and its association with conduction abnormalities. Including controlled hypertension‐only cohorts or normotensive HFpEF models would be invaluable for delineating the specific contributions of pressure overload. Additionally, investigating the effects of standard‐of‐care therapies (e.g., SGLT2 inhibitors) on the identified pathways represents a crucial and logical next step, which would substantially enhance the clinical relevance of these findings.

### 4.2 Conclusion

By applying an optimized STE protocol specifically tailored for the left atrium, this study markedly improves the precision of atrial functional assessment. Unlike conventional metrics that primarily focused on LV diastolic filling, we demonstrate that LASI serves as a sensitive and integrative marker, capturing both AV compliance and underlying structural remodelling. These findings not only enhance mechanistic understanding of atrial cardiomyopathy but also highlight the translational potential of LASI‐ and CSRe‐based parameters as non‐invasive surrogates for tissue‐level remodelling, with implications for early HFpEF diagnosis, refined phenotyping, and monitoring of therapeutic response.

## AUTHOR CONTRIBUTIONS

Qingfeng Zhang was responsible for conceptualization, formal analysis, investigation, data curation, original draft preparation, project administration, and funding acquisition. Wenhua Li contributed to conceptualization, resources, review and editing, visualization, and funding acquisition. Yi Wang supervised the study and contributed to methodology. Hongmei Zhang was responsible for software development, and Lixue Yin performed data validation. All authors have read and approved the final version of this manuscript and agree to be accountable for all aspects of the work in ensuring that questions related to the accuracy or integrity of any part of the work are appropriately investigated and resolved. All persons designated as authors qualify for authorship, and all those who qualify for authorship are listed.

## CONFLICT OF INTEREST

None declared.

## Data Availability

The datasets generated and/or analyzed during the current study are available from the corresponding author upon reasonable request.
